# Supplementary material for: Transfer learning enables identification of multiple types of RNA modifications using nanopore direct RNA sequencing
Source: Nat Commun. 2024 May 14;15:4049. doi: 10.1038/s41467-024-48437-4 (PMC11094168; doi:10.1038/s41467-024-48437-4)
Supplement: Supplementary file 1 — Supplementary Information File [file 41467_2024_48437_MOESM1_ESM.pdf]

Transfer learning enables identification of multiple types of RNA modifications using nanopore direct RNA sequencing

You Wu[1], Wenna Shao[1], Mengxiao Yan[2], Yuqin Wang[2], Pengfei Xu[1], Guoqiang Huang[1], Xiaofei Li[1], Brian D. Gregory[3], Jun Yang[2,4,*], Hongxia Wang[2,4,*], Xiang Yu[1,*]

[1]Joint International Research Laboratory of Metabolic & Developmental Sciences, School of Life Sciences and Biotechnology, Shanghai Jiao Tong University, Shanghai 200240, China

[2]Shanghai Key Laboratory of Plant Functional Genomics and Resources, Shanghai Chenshan Botanical Garden, Shanghai 201602, China

[3]Department of Biology, University of Pennsylvania, Philadelphia, PA 19104, USA

[4]Chenshan Scientific Research Center of CAS Center for Excellence in Molecular Plant Sciences, Shanghai 201602, China

*Corresponding author: Xiang Yu, yuxiang2021@sjtu.edu.cn; Hongxia Wang, hxwang@cemps.ac.cn; Jun Yang, jyang03@cemps.ac.cn.

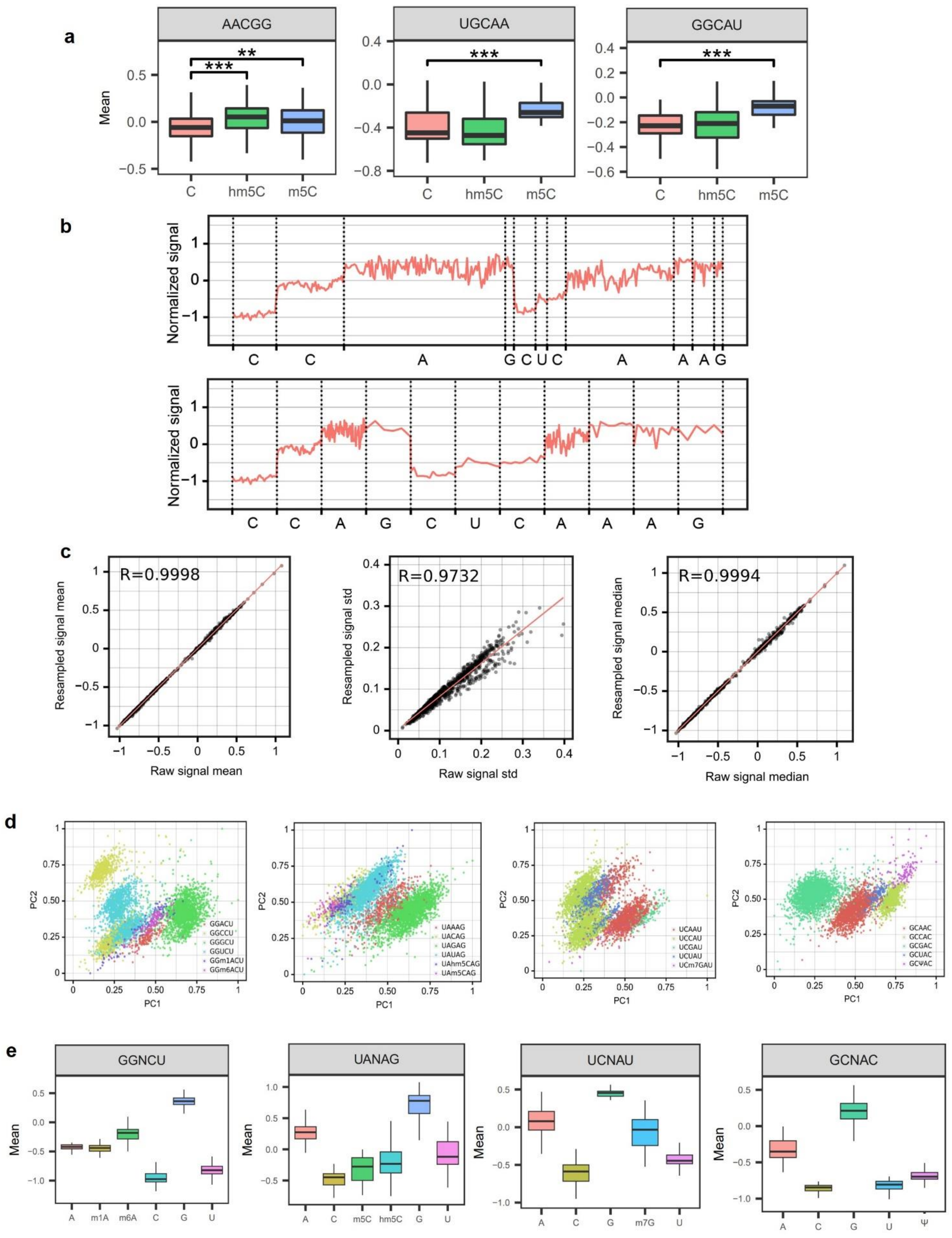

Supplementary Fig. 1: DRS features at base level and current level affected by 5-mer sequence contexts. **a,** Mean signatures of current signals at C, hm^{5}C and m^{5}C under different 5-mer sequence contexts. The upper and lower limits represent the 75th and 25th percentiles, respectively, while the center line represents the

median; upper and lower whiskers indicate ±1.5× the interquartile range. Outliers are not shown in these figures. All statistical tests used two-sided Wilcoxon tests. Significance levels are: *$p < 0.05$, **$p < 0.01$, ***$p < 0.001$. The exact p-values are provided in the Source Data file. **b,** An example showing normalized current signals with raw signal width (top panel), and resampled current signals with equal width (bottom panel). **c,** Scatterplot of base-level features including mean, std and median between original signals and resampled signals. R value indicates the correlation coefficient. **d,** Scatter plots displaying the difference between the modified bases and the four canonical bases after PCA transform, with the two bases before and after the target base being the same. **e**, The mean feature between the modified bases and the four canonical bases. The upper and lower limits represent the 75th and 25th percentiles, respectively, while the center line represents the median; upper and lower whiskers indicate ±1.5× the interquartile range. Outliers are not shown in these figures. Source data are provided as a Source Data file.

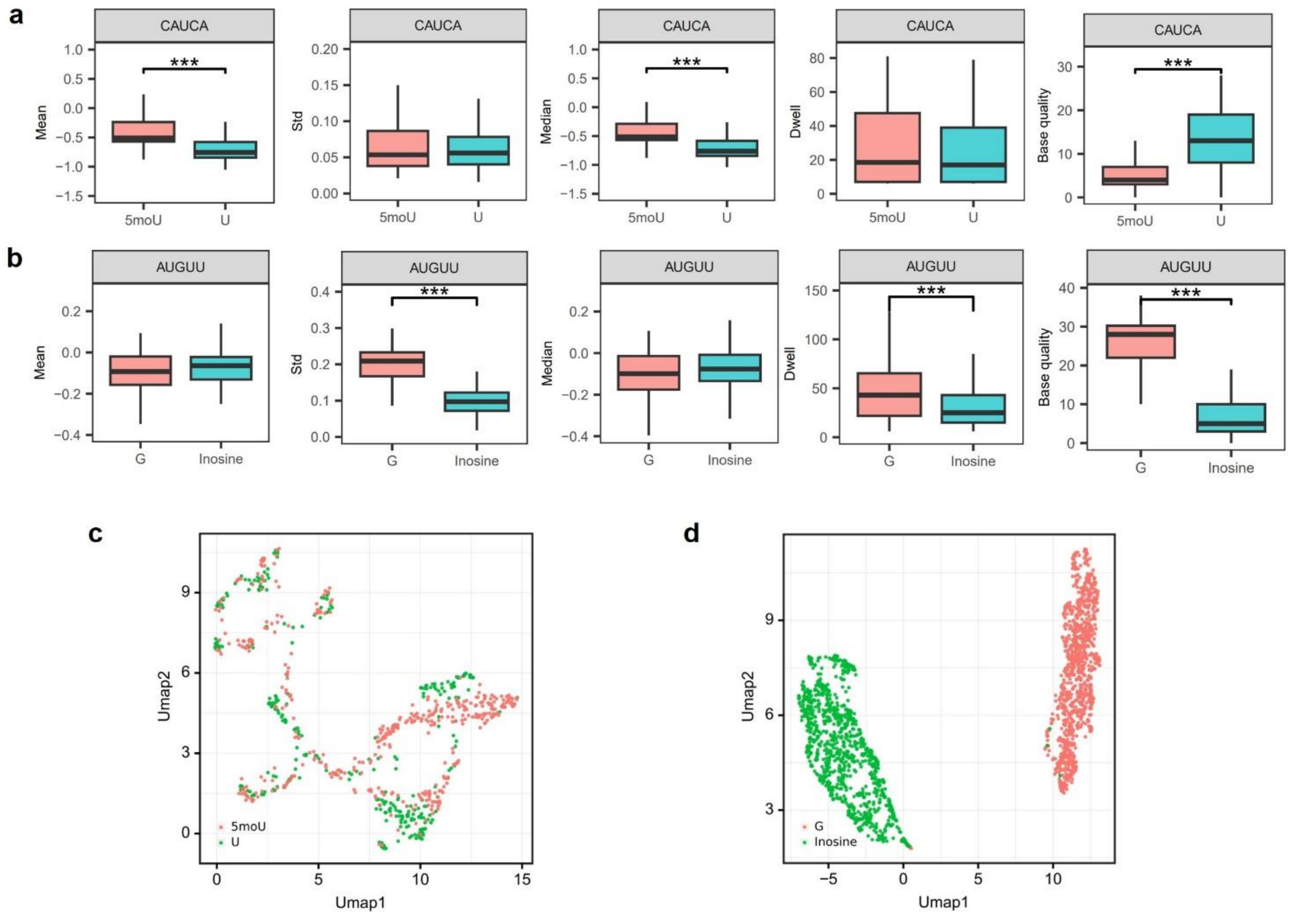

Supplementary Fig. 2: DRS features at base level and current level of 5moU and Inosine from the ELIGOS datasets. a, Base-level features (mean, std, median, dwell and base quality) of 5moU compared to normal U base. The upper and lower limits represent the 75th and 25th percentiles, respectively, while the center line represents the median; upper and lower whiskers indicate ±1.5× the interquartile range. Outliers are not shown in these figures. All statistical tests used two-sided Wilcoxon tests. Significance levels are: *$p < 0.05$, **$p < 0.01$, ***$p < 0.001$. The exact p-values are provided in the Source Data file. **b**, Base-level features (mean, std, median, dwell and base quality) of Inosine compared to G. The upper and lower limits represent the 75th and 25th percentiles, respectively, while the center line represents the median; upper and lower whiskers indicate ±1.5× the interquartile range. Outliers are not shown in these figures. All statistical tests used two-sided Wilcoxon tests. Significance levels are: *$p < 0.05$, **$p < 0.01$, ***$p < 0.001$. The exact p-values are provided in the Source Data file. **c**, Umap visualization of resampled current signals of 5moU and U under the sequence context CAUCA. **d**, Umap visualization of resampled current signals of Inosine and G under the sequence context CAUCA. Source data are provided as a Source Data file.

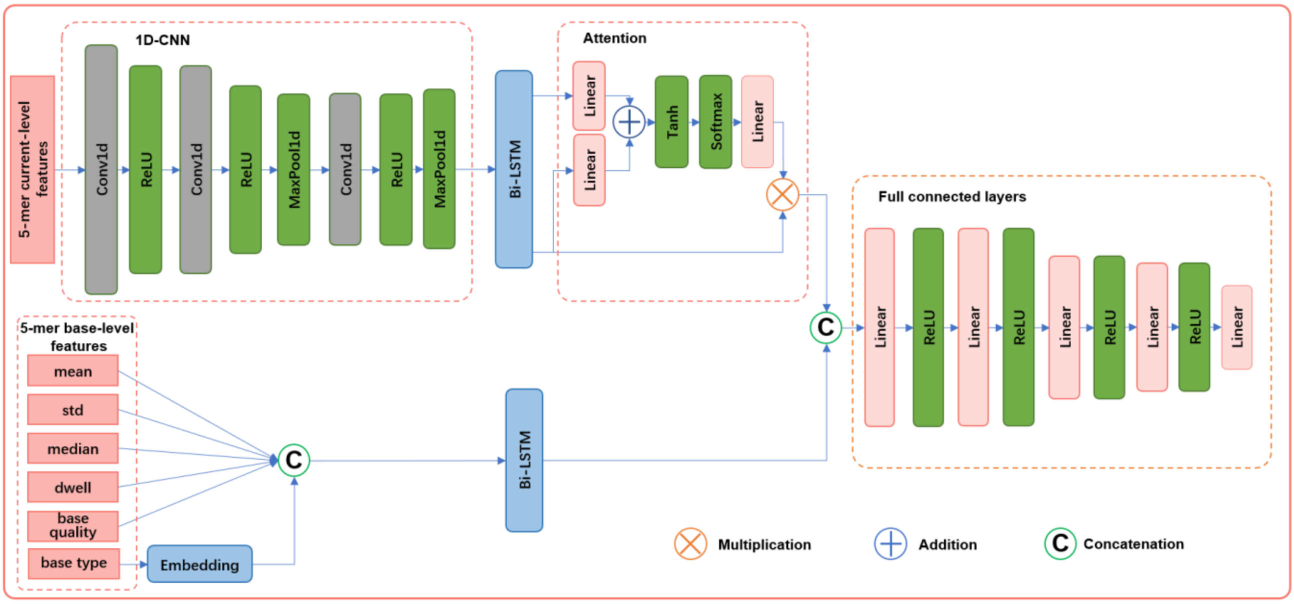

Supplementary Fig. 3: Detailed architecture of TandemMod model. TandemMod consists of 4 main components: a one-dimensional convolutional neural network(1D-CNN), a bi-directional long short-term memory (bi-LSTM) module, an attention mechanism and full-connected layers. The top layers of the model act as feature extractor that learns modification-related information and the bottom layers act as classifier that predict modification type.

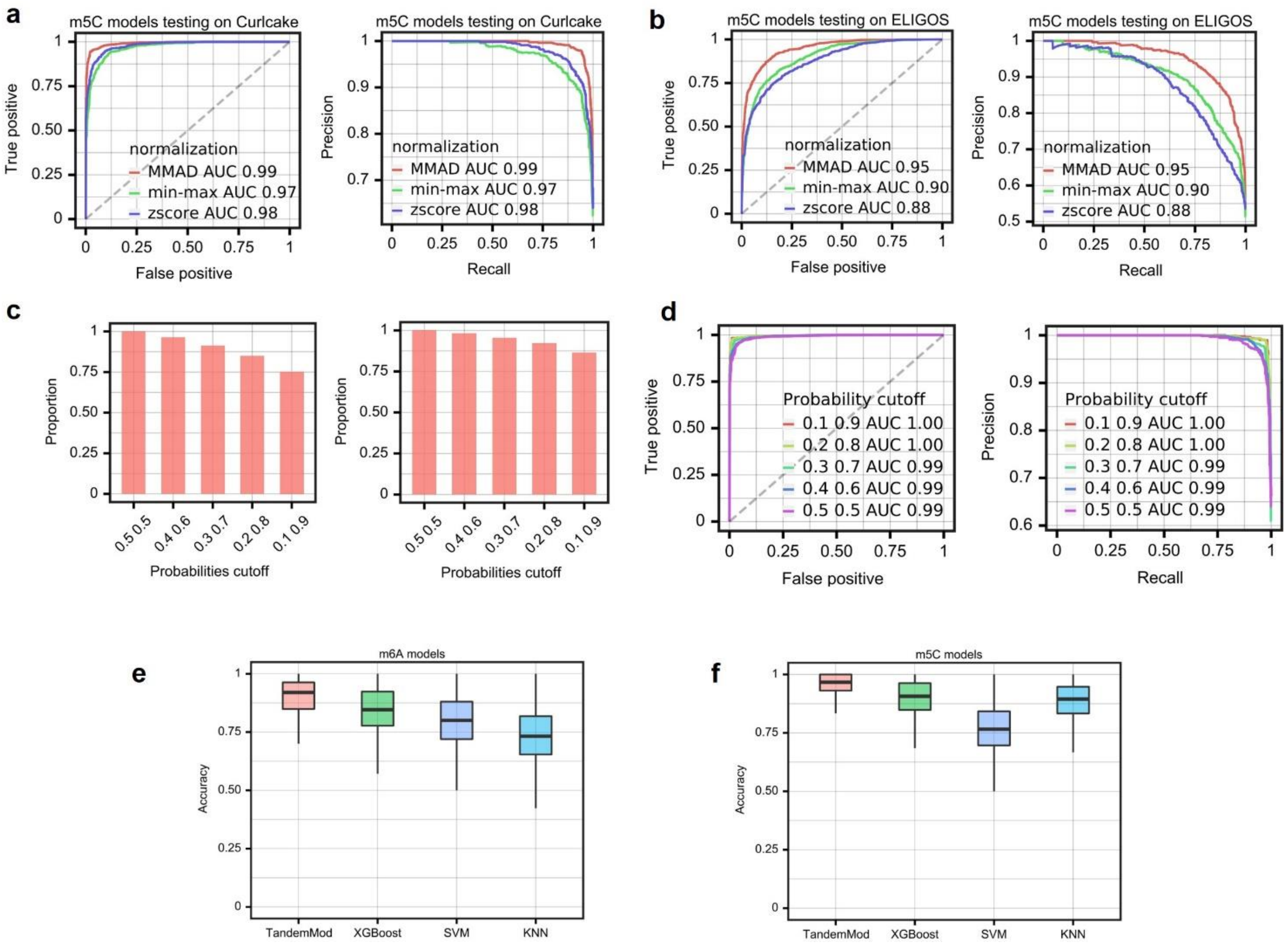

Supplementary Fig. 4: Performance evaluation of TandemMod. a, ROC curve and PR curve showing the performance for m^5C identification on Curlcake testing set normalized with z-score, min-max scaling or MMAD. **b,** ROC curve and PR curve showing the performance for m^5C identification on ELIGOS testing set normalized with z-score, min-max scaling or MMAD. **c,** Relationship between the remaining reads and the cutoff probabilities of ELIGOS (left panel) and Curlcake (right panel). **d,** ROC curve and PR curve showing the performance for m^5C identification model on Curlcake dataset using different cutoff values. **e,** Comparison of models trained on Curlcake m^5C dataset and tested on Curlcake testing set. The upper and lower limits represent the 75th and 25th percentiles, respectively, while the center line represents the median; upper and lower whiskers indicate ±1.5× the interquartile range. Outliers are not shown in these figures. **f,** Comparison of models trained on Curlcake m^6A dataset and tested on Curlcake testing set. The upper and lower limits represent the 75th and 25th percentiles, respectively, while the center line represents the median; upper and lower whiskers indicate ±1.5× the interquartile range. Outliers are not shown in these figures. Source data are provided as a Source Data file.

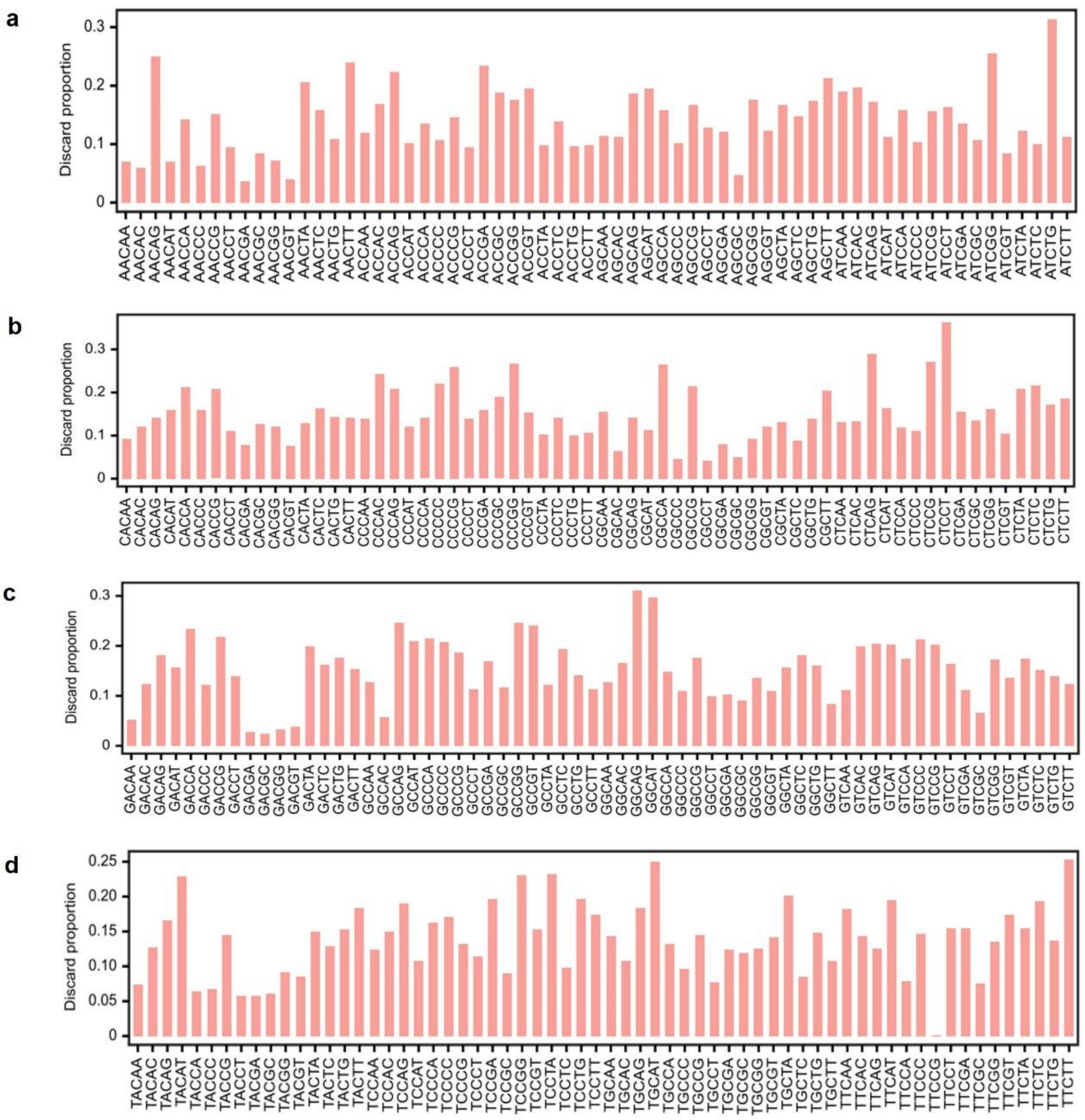

Supplementary Fig. 5: The proportion of discarded reads for the 256 motifs when applying a probability cutoff strategy with the threshold of 0.3-0.7. a-d, The distribution of discarded reads was found to be uniform across all motifs. Source data are provided as a Source Data file.

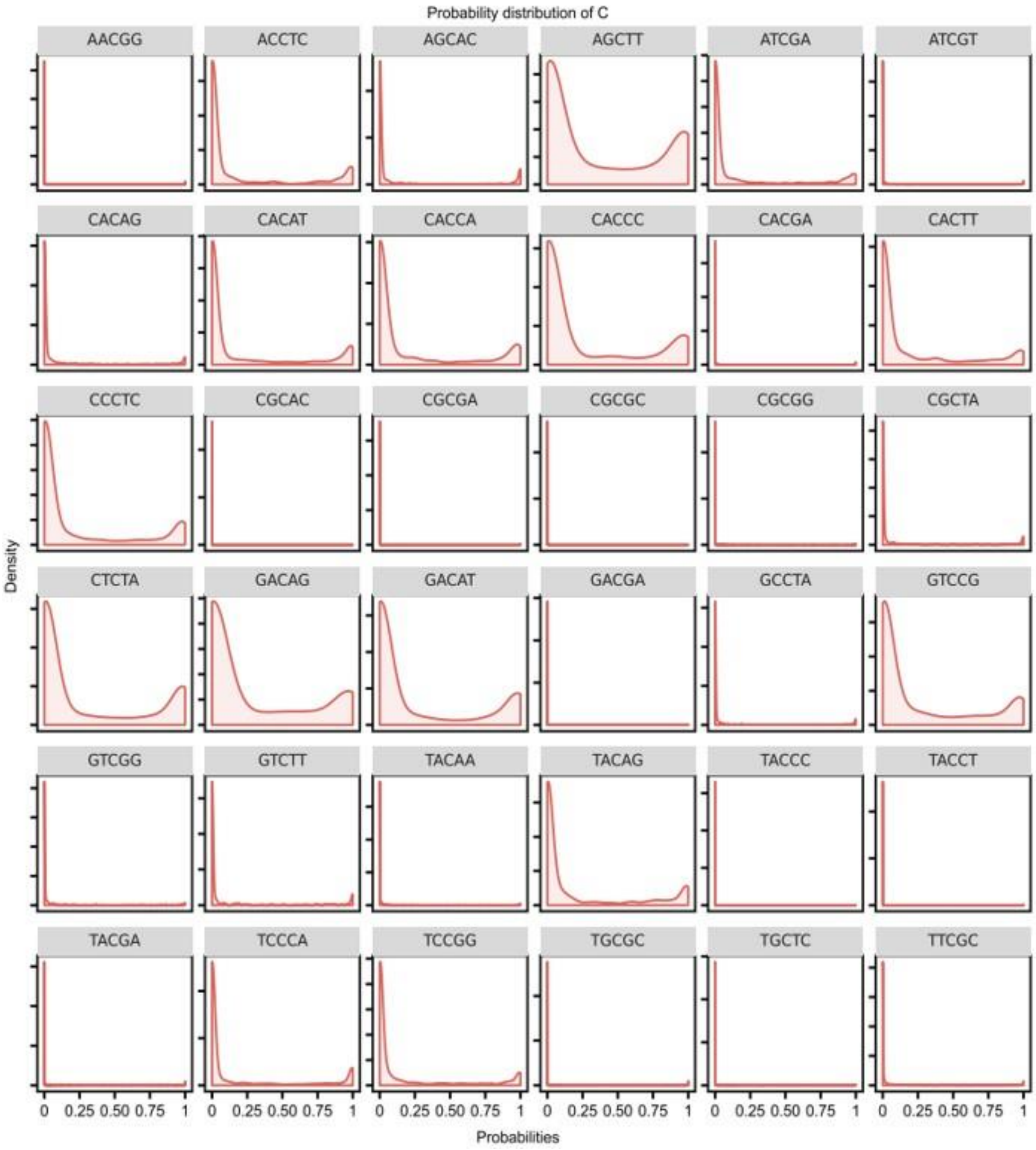

Supplementary Fig. 6: The predicted modification probabilities distribution for ELIGOS normal C sites. The 36 motifs were randomly selected from the 256 motifs using seed 0 with the random package in Python. Source data are provided as a Source Data file.

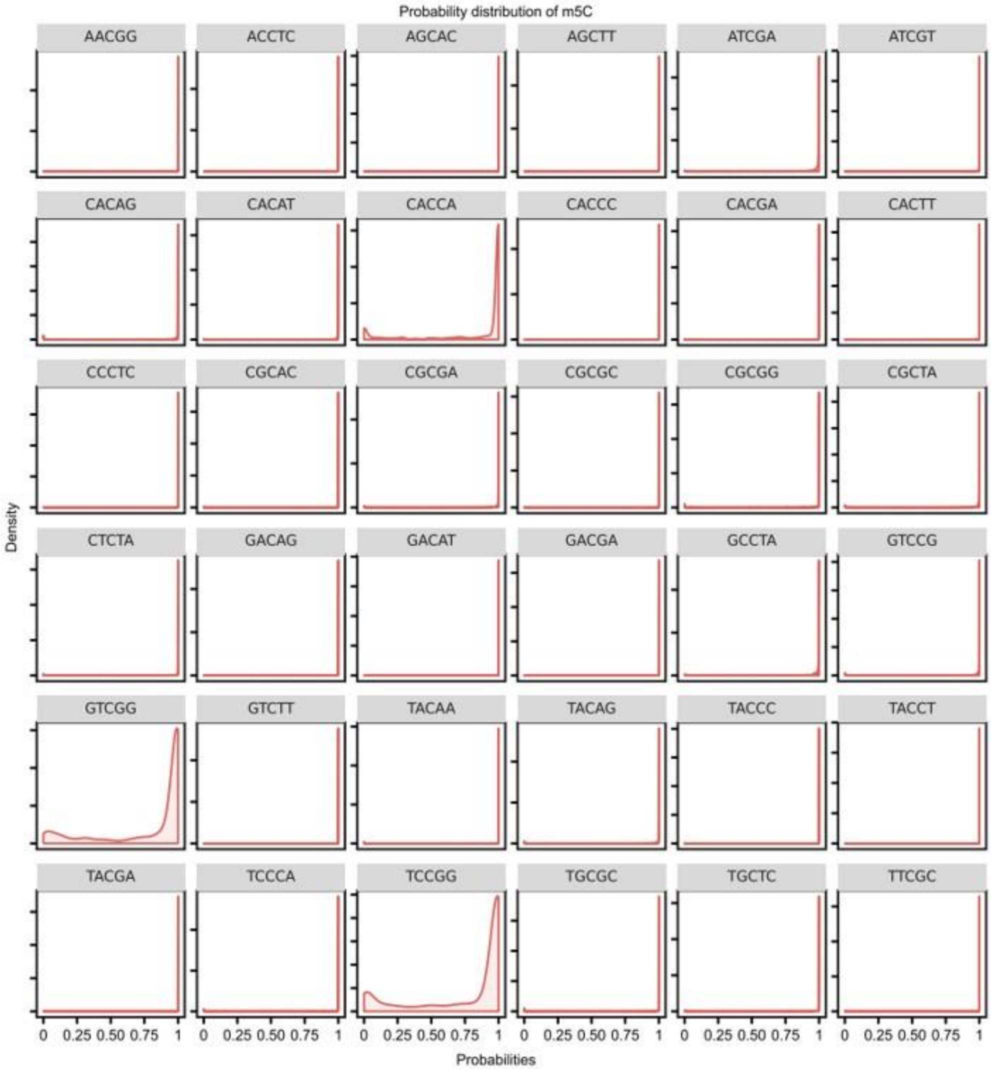

Supplementary Fig. 7: The predicted modification probabilities distribution for ELIGOS m5C sites. The 36 motifs were randomly selected from the 256 motifs using seed 0 with the random package in Python. Source data are provided as a Source Data file.

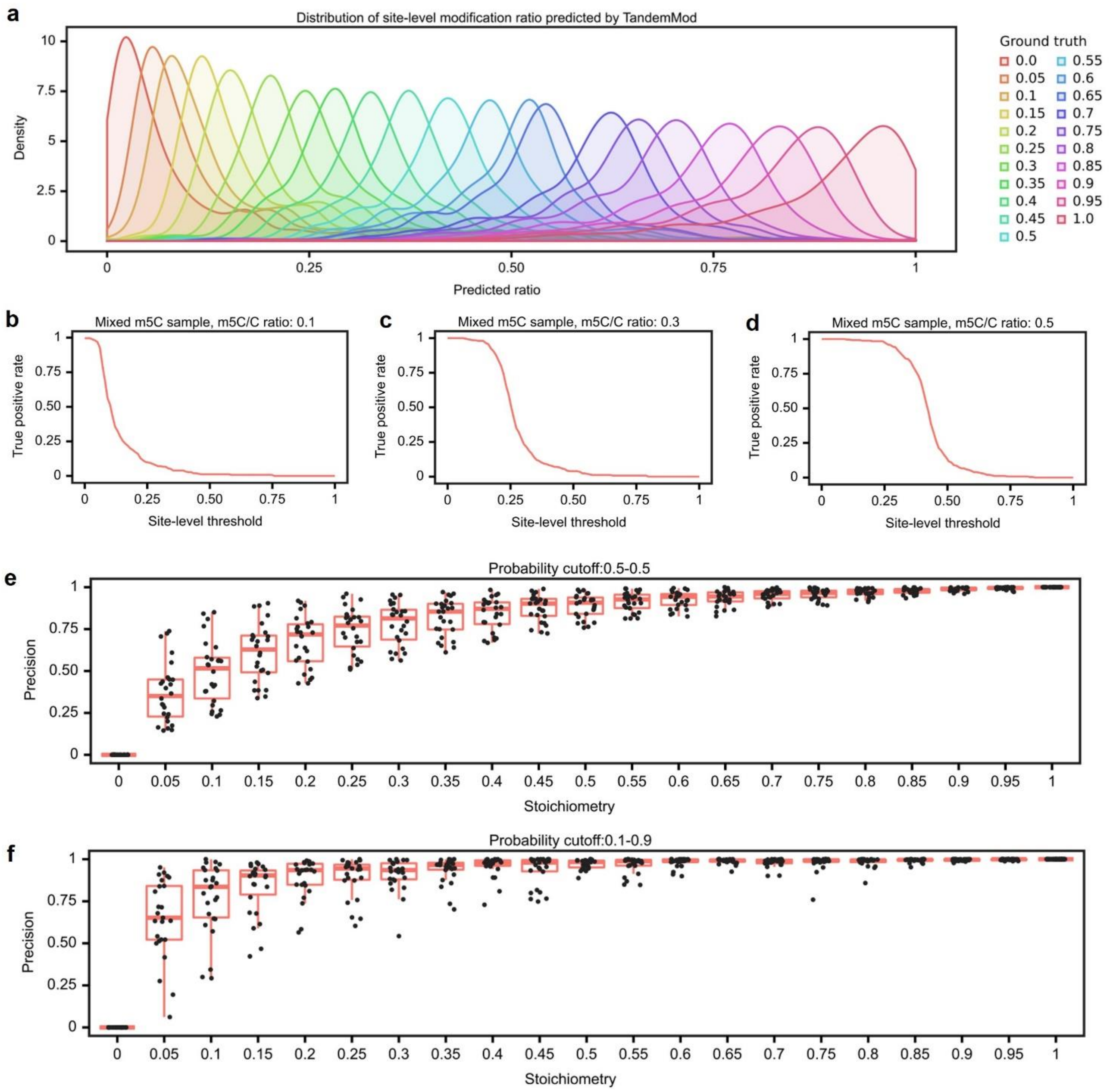

Supplementary Fig. 8: Site-level performance evolution of TandemMod on mixed m5C samples. **a**, The predicted site-level modification rates gradually increase align well with the ground truth stoichiometry increasing. Predicted site-level modification rates under various stoichiometries. The datasets used in this analysis were mixed from the ELIGOS-m^{5}C dataset and the ELIGOS-normal C datasets. The distribution shows a gradual increase in predicted modification rates, aligning closely with the ground truth stoichiometry. **b**, The influence of site-level cutoff threshold on the true positive rate in the sample with m5C/C ratio of 0.1. **c**, The influence of site-level cutoff threshold on the true positive rate in the sample with m5C/C ratio of 0.3. **d**, The influence of site-level cutoff threshold on the true positive rate in the sample with m5C/C ratio of 0.5. **e**, The model precision on the mixed samples with probability cutoff of 0.5-0.5. The precision is low where the sample is unbalanced. The upper and lower limits represent the 75th and 25th percentiles, respectively, while the center line represents the median; upper and lower whiskers indicate ±1.5× the interquartile range. **f**, The model precision on the mixed samples with probability cutoff of 0.1-0.9. The precision was significantly improved, indicating the effectiveness of applying a higher cutoff value. The upper and lower limits represent the 75th

and 25th percentiles, respectively, while the center line represents the median; upper and lower whiskers indicate ±1.5× the interquartile range. Source data are provided as a Source Data file.

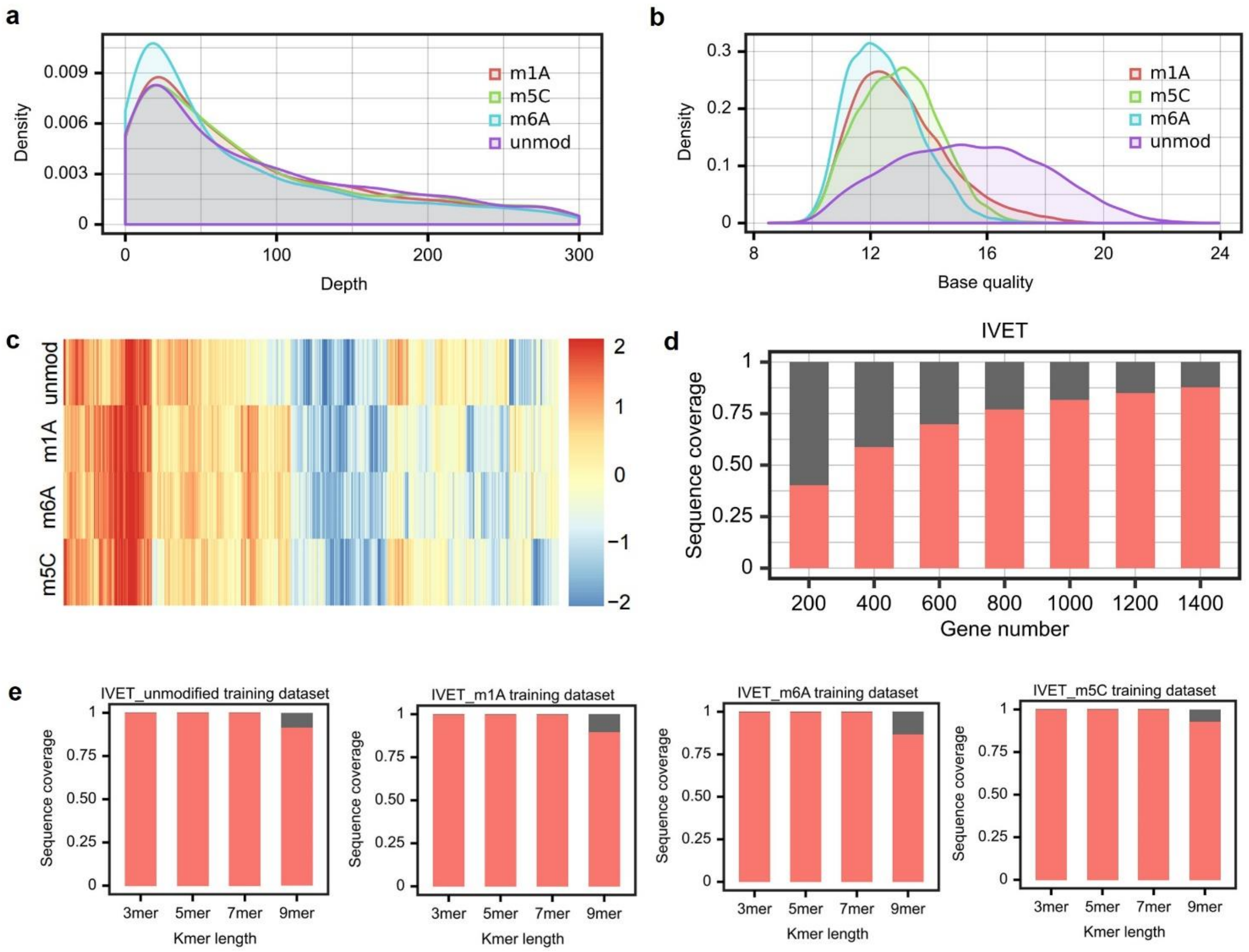

Supplementary Fig. 9: Statistics of direct RNA sequencing results from the IVET datasets. a, The sequencing depth of the four IVET datasets. **b,** The mean base quality of the four IVET datasets. **c**, Heatmap displaying the transcribed mRNA abundance across the four IVET samples. **d**, The barplot showing the 9-mer sequence coverage across different numbers of genes in the IVET dataset. **e**, The sequence coverage of the four IVET training data. Source data are provided as a Source Data file.

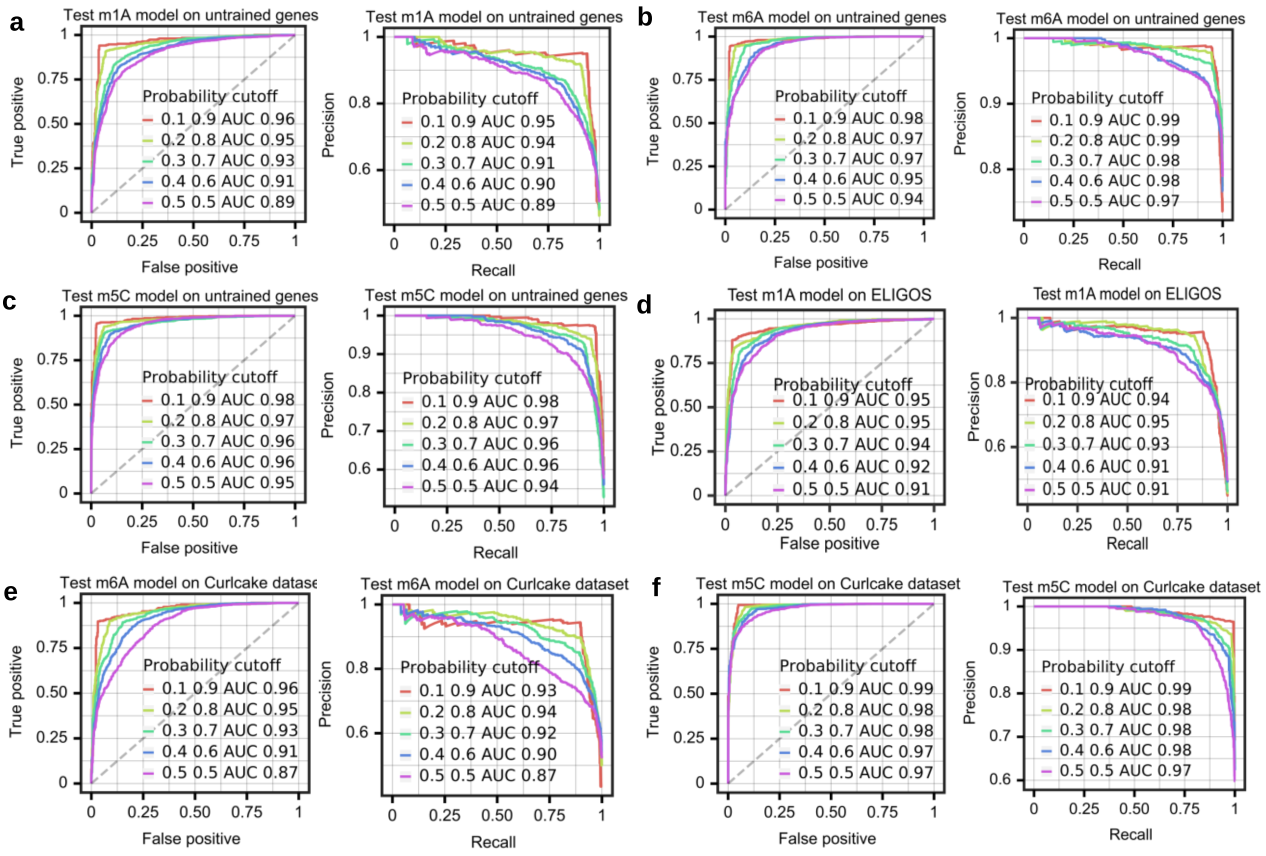

Supplementary Fig. 10: Performance evaluation of TandemMod models trained on IVET datasets. a-c, ROC curve and PR curve showing the performance evaluation of the m^1A model (**a**), m^6A model (**b**) and m^5C model (**c**) trained on the IVET training sets and tested on the IVET independent genes. **d,** ROC curve and PR curve showing the performance evaluation of the m^1A model trained on the IVET training set and tested on ELIGOS dataset. **e,** ROC curve and PR curve showing the performance evaluation of the m^6A model trained on the IVET training set and tested on the Curlcake dataset. **f,** ROC curve and PR curve showing the performance evaluation of the m^5C model trained on the IVET training set and tested on the Curlcake dataset.

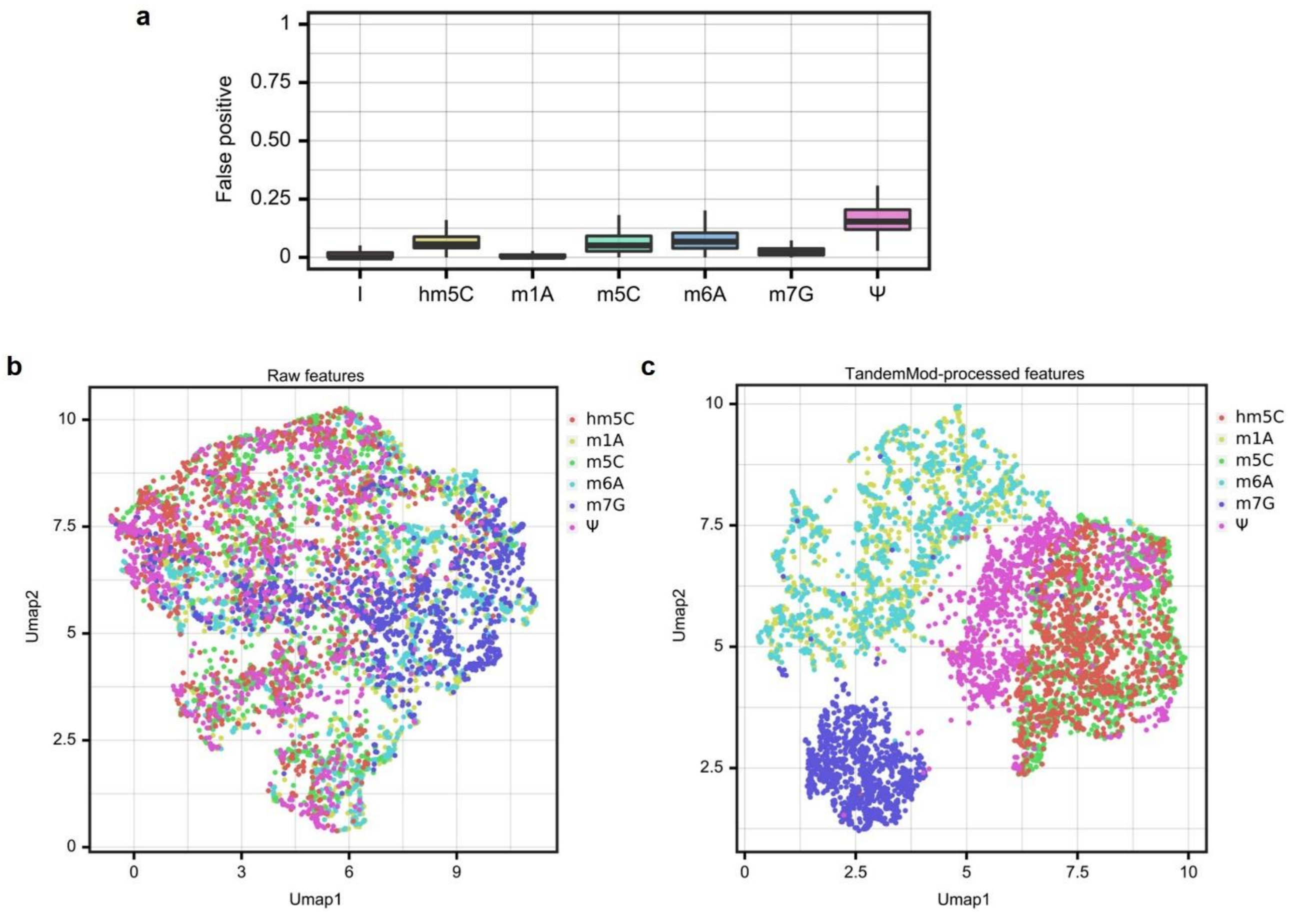

Supplementary Fig. 11: Multiple types of RNA modification. a, Boxplot showing false positive rates of the 7 modification detection models testing on IVET modification-free dataset. The upper and lower limits represent the 75th and 25th percentiles, respectively, while the center line represents the median; upper and lower whiskers indicate ±1.5× the interquartile range. Outliers are not shown this figure. **b,** Umap visualization of raw features of the 6 modifications in ELIGOS datasets. **c**, Umap visualization of TandemMod-processed features of the 6 modifications in ELIGOS datasets. Source data are provided as a Source Data file.

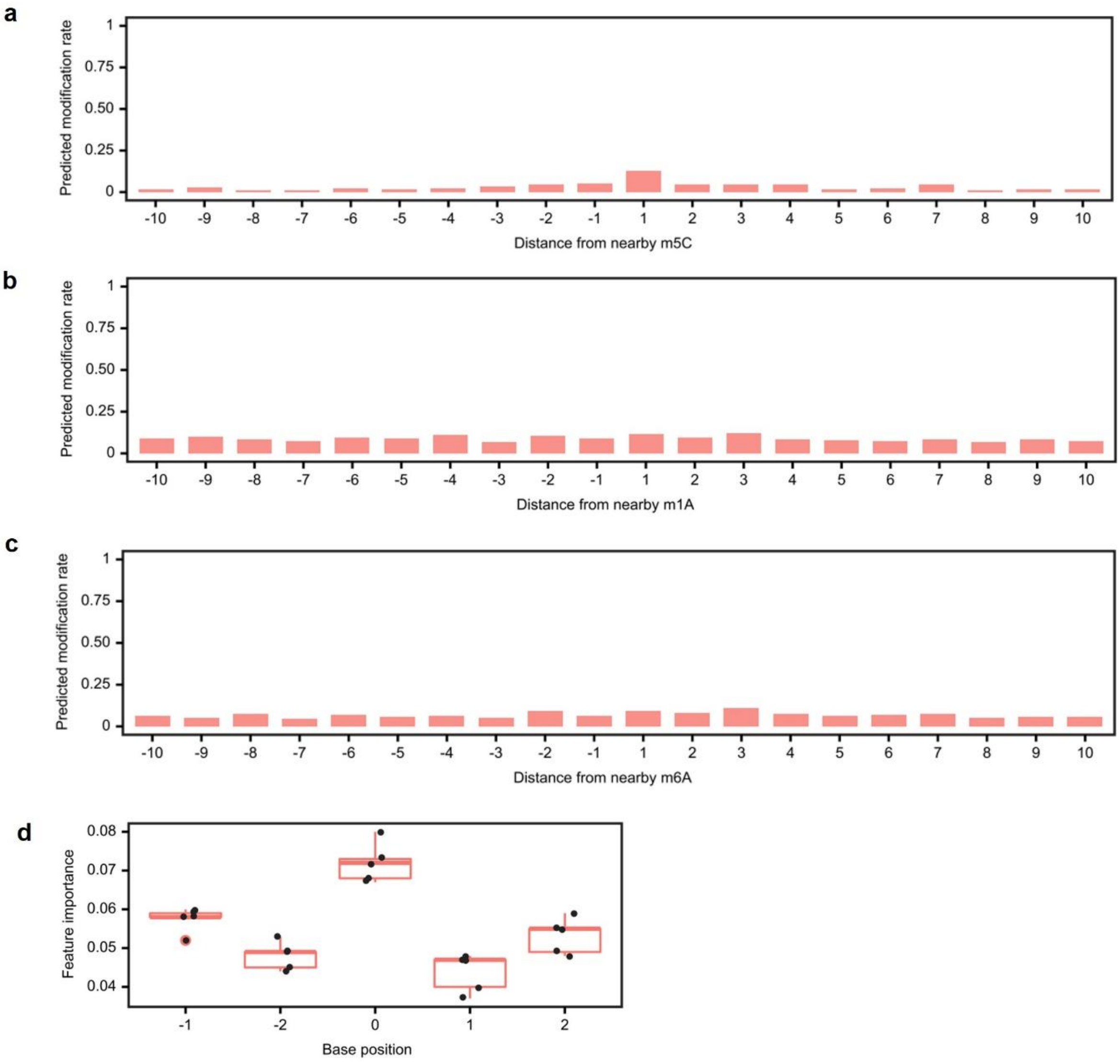

Supplementary Fig. 12: Impact of other modifications on the identification of m^{6}A and m^{5}C in IVET datasets. a, Test the TandemMod-m^{6}A model on A sites from the IVET-m^{5}C dataset. We selected reads at different distances (-10 to 10) from m^{5}C, predicted the modification probabilities of these A sites, and found that false positives were minimally influenced by neighboring modified sites. **b,** Test the TandemMod-m^{5}C model on C sites from the IVET-m^{1}A dataset. **c,** Test the TandemMod-m^{5}C model on C sites from the IVET-m^{6}A dataset. **d,** The feature importance of the five consecutive bases learned by TandemMod. The results showed that although TandemMod takes 5-mer motif features as input, it focuses more on the central bases than on the flanking bases. This characteristic accounts for that TandemMod is not significantly influenced by neighboring modified bases. The upper and lower limits represent the 75th and 25th percentiles, respectively, while the center line represents the median; upper and lower whiskers indicate ±1.5× the interquartile range. Source data are provided as a Source Data file.

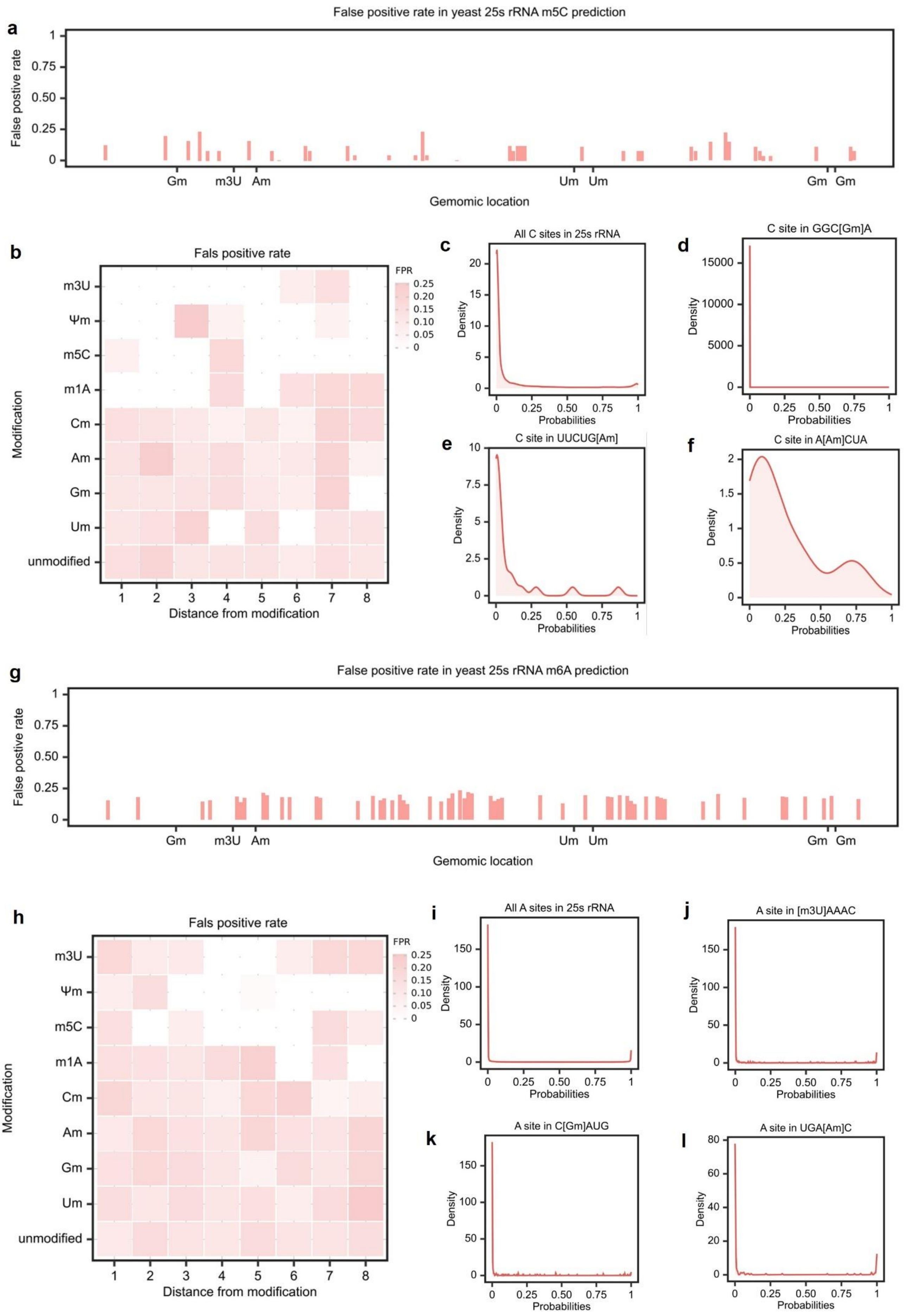
a
False positive rate in yeast 25s rRNA m5C prediction
False postive rate
Gm m3U Am Um Um Gm Gm
Gemomic location
b
Fals positive rate
FPR
m3U
Ψm
m5C
m1A
Cm
Am
Gm
Um
unmodified
Modification
Distance from modification
c
All C sites in 25s rRNA
d
C site in GGC[Gm]A
e
C site in UUCUG[Am]
f
C site in A[Am]CUA
Density
Probabilities
g
False positive rate in yeast 25s rRNA m6A prediction
False postive rate
Gm m3U Am Um Um Gm Gm
Gemomic location
h
Fals positive rate
FPR
Modification
Distance from modification
i
All A sites in 25s rRNA
j
A site in [m3U]AAAC
k
A site in C[Gm]AUG
l
A site in UGA[Am]C
Density
Probabilities

Supplementary Fig. 13: Impact of other modifications on the identification of m^6A and m^5C. **a**, Barplot showing overall false positive rate of m^5C predictions in yeast 25s rRNA. **b**, The impact of the proximity of nearby modifications on the false positive rate of m^5C predictions. The results indicate that the false positive rate did not increase in the presence of neighboring modifications. **c**, The prediction probability distribution in of all C sites in yeast 25s rRNA. **d**, The prediction probability distribution of C site with nearby Gm modification (GGC[Gm]A motif). **e**, The prediction probability distribution of C site with nearby Am modification (UUCUG[Am] motif). **f**, The prediction probability distribution of C site with nearby Am modification (A[Am]CUA motif). **g**, Barplot showing overall false positive rate of m^6A predictions in yeast 25s rRNA. **h**, The impact of the proximity of nearby modifications on the false positive rate of m^6A predictions. The results indicate that the false positive rate did not increase in the presence of neighboring modifications. **i**, The prediction probability distribution in of all A sites in yeast 25s rRNA. **j**, The prediction probability distribution of A site with nearby Gm modification (UGA[Am]C motif). **k**, The prediction probability distribution of A site with nearby Am modification (C[Gm]AUG motif). **l**, The prediction probability distribution of A site with nearby Am modification ([m^3U]AAAC motif).

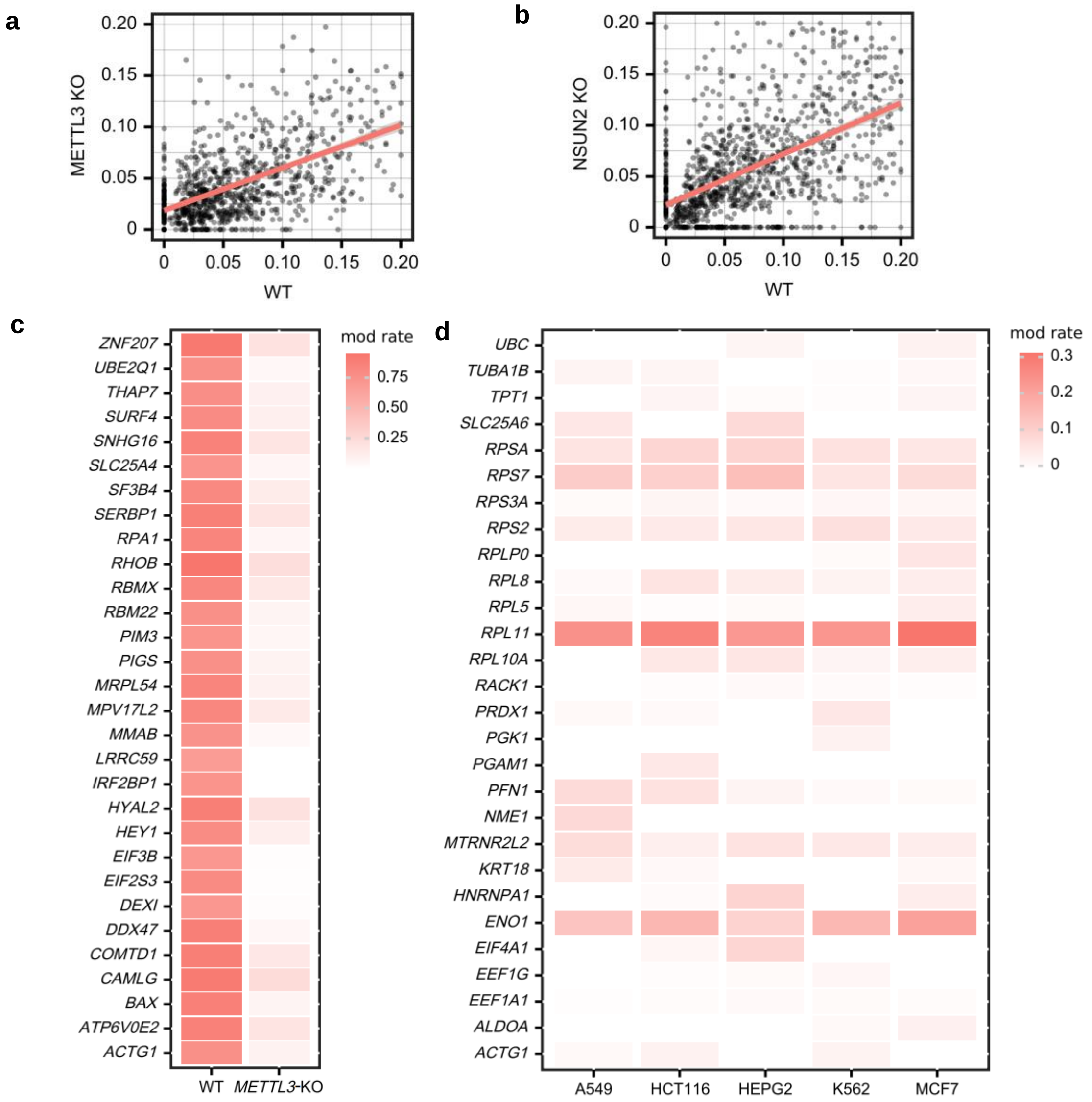

Supplementary Fig. 14: Identification of m^{6}A sites across different tissues in human. **a**, Scatter plot showing decreased m^{6}A level in *METTL3*-KO HEK293T sample compared to WT sample. **b**, Scatter plot showing decreased m^{5}C level in *NSUN2*-KO HeLa sample compared to WT sample. **c**, Heatmap of top 30 genes with differentially m^{6}A-modified rates between *METTL3*-KO and WT samples. **d**, Heatmap showing m^{6}A rates of randomly selected genes across 5 human cell lines. Source data are provided as a Source Data file.

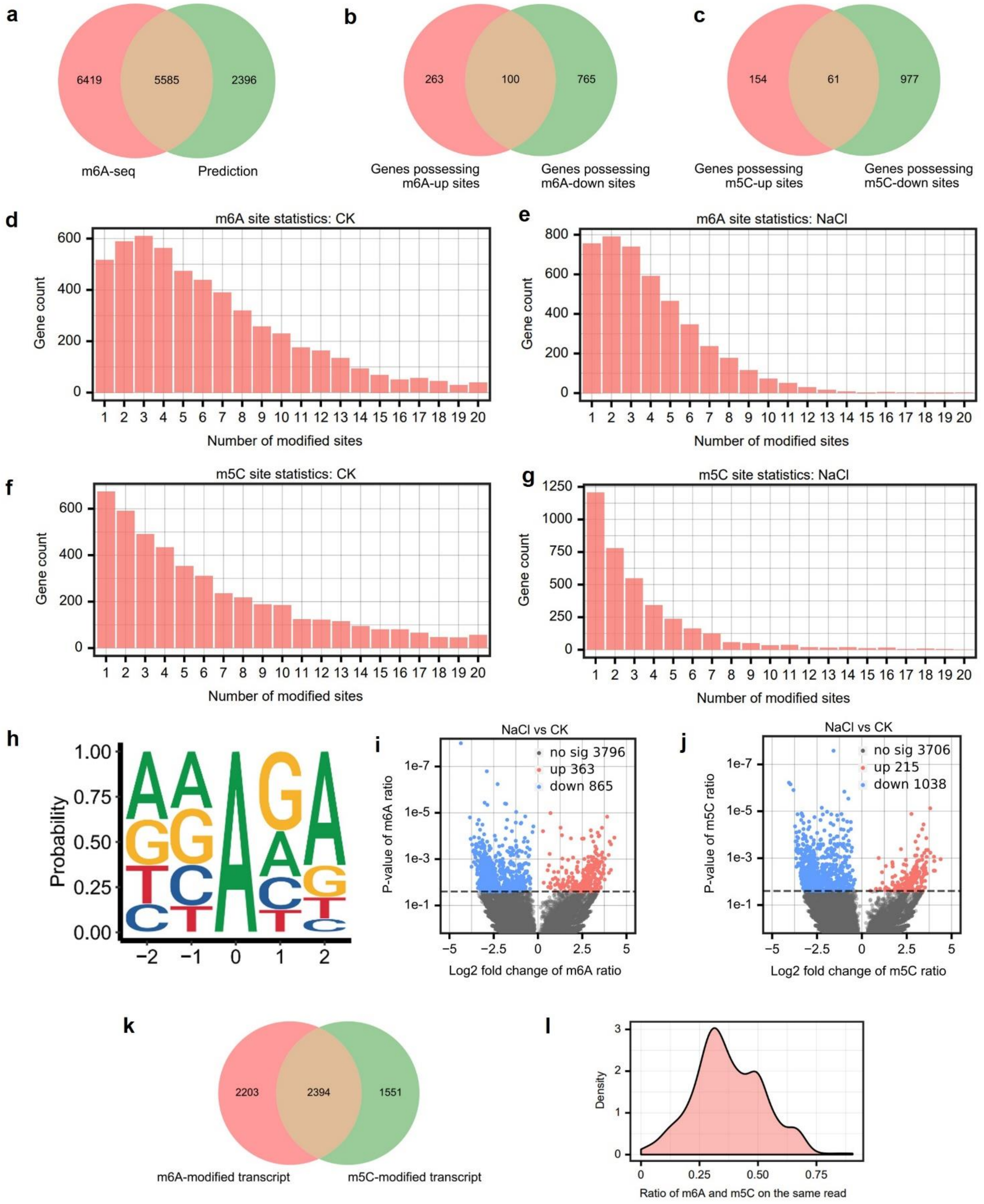

Supplementary Fig. 15: Identification of m^6A and m^5C sites across different conditions in rice. a, Venn diagram showing gene with TandemMod-predicted m^6A sites and genes detected by m^6A-seq. **b**, Venn diagram of genes containing m^6A sites with increased and decreased modification rate. **c**, Venn diagram showing of genes containing m^5C sites with increased and decreased modification rate. **d-e,** Statistics of genes possessing different number of m^6A-modified sites in CK (**d**) and NaCl-treated sample (**e**). **f-g,**

Statistics of genes possessing different number of m^5C-modified sites in CK (**f**) and NaCl-treated sample (**g**). **h**, Sequence motif of m^6A-modified sites enriched in rice control sample in addition to DRACH. **i**, Volcano plot showing differentially m^6A-modified genes. **j**, Volcano plot showing differentially m^5C-modified genes. Chi-square test was performed and sites with Chi-square p-value less than 0.05 were considered as differentially modified. **k**, Venn diagram showing overlap mRNAs with co-occurrence of m6A and m5C modification sites. **l,** The frequency of m6A and m5C co-occurrence at the same long transcript for each gene. Source data are provided as a Source Data file.

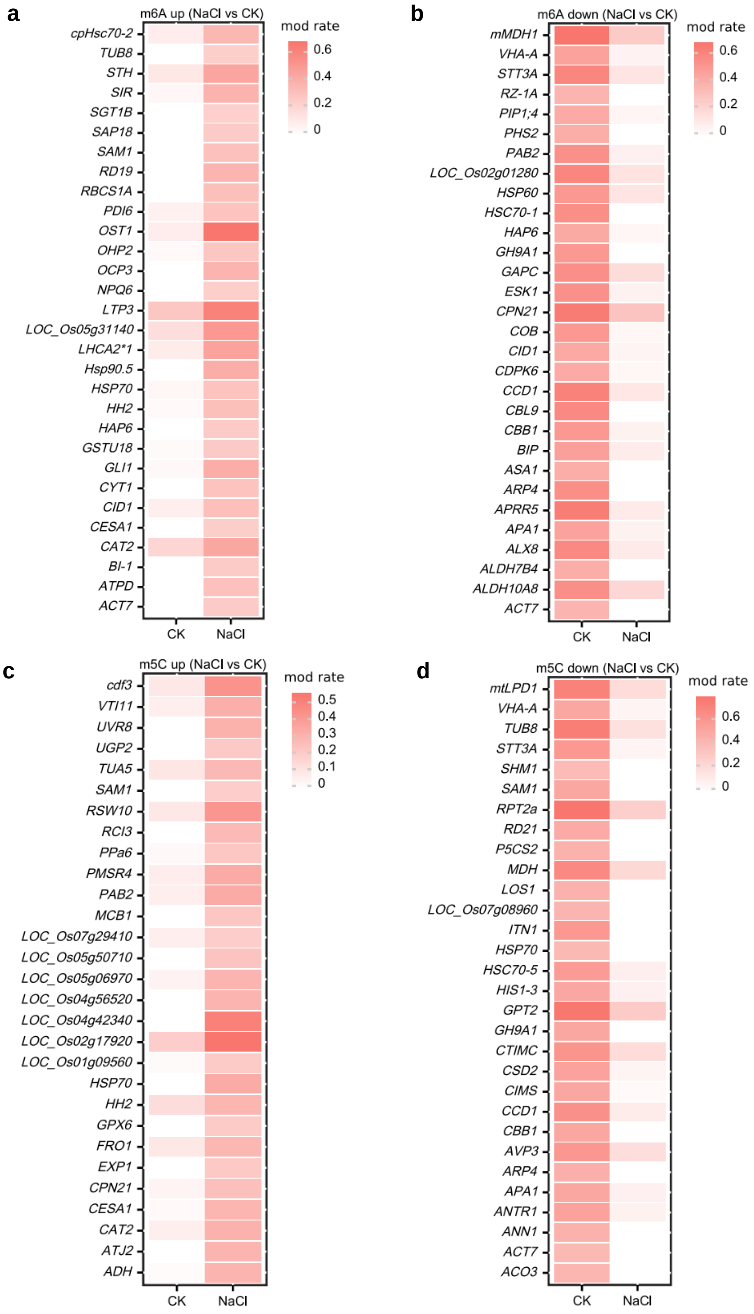

Supplementary Fig. 16: Differentially m^{6}A-modified and m^{5}C-modified genes identified by TandemMod in NaCl-treated rice as compared to the control. a, Genes with increased m^{6}A modification rate in NaCl-treated sample. **b**, Genes with decreased m^{6}A modification rate in NaCl-treated sample. **c**, Genes with increased m^{5}C modification rate in NaCl-treated sample. **d**, Genes with decreased m^{5}C modification rate in NaCl-treated sample.
