# Supplementary material for: Transfer learning enables identification of multiple types of RNA modifications using nanopore direct RNA sequencing
Source: Nat Commun. 2024 May 14;15:4049. doi: 10.1038/s41467-024-48437-4 (PMC11094168; doi:10.1038/s41467-024-48437-4)
Supplement: Supplementary file 3 — Description of Additional Supplementary Files [file 41467_2024_48437_MOESM3_ESM.pdf]

Description of Additional Supplementary Files:

Supplementary Data 1: m^6A-modified sites detected by TandemMod in rice control sample.

Supplementary Data 2: m^5C-modified sites detected by TandemMod in rice control sample.

Supplementary Data 3: Genes with differential m^6A-modified rates detected by TandemMod in rice sample with NaCl treatment as compared to control. Two-sided Fisher's exact test was used without adjusting for multiple comparisons.

Supplementary Data 4: Genes with differential m^5C-modified rates detected by TandemMod in rice sample with NaCl treatment as compared to control. Two-sided Fisher's exact test was used without adjusting for multiple comparisons.
